# Comparison of Vitamin D and 25-Hydroxyvitamin D Concentrations in Human Breast Milk between 1989 and 2016–2017

**DOI:** 10.3390/nu13020573

**Published:** 2021-02-09

**Authors:** Naoko Tsugawa, Mayu Nishino, Akiko Kuwabara, Honami Ogasawara, Maya Kamao, Shunjiro Kobayashi, Junichi Yamamura, Satoshi Higurashi

**Affiliations:** 1 Department of Health and Nutrition, Osaka Shoin Women’s University, Higashiosaka, Osaka 577-8550, Japan; ogasawara.honami@osaka-shoin.ac.jp; 2 Division of Human Dietics, Graduate School of Human Science, Osaka Shoin Women’s University, Higashiosaka, Osaka 577-8550, Japan; laugh_xx5m2a9y6u.com@ezweb.ne.jp; 3 Department of Clinical Nutrition, Faculty of Comprehensive Rehabilitation, Osaka Prefecture University, Habikino, Osaka 583-8555, Japan; akuwabara@rehab.osakafu-u.ac.jp; 4 Extension Center, Kobe Pharmaceutical University, Kobe, Hyogo 658-8558, Japan; m-kamao@kobepharma-u.ac.jp; 5 Department of Research & Development, Bean Stalk Snow Co., Ltd., Kawagoe, Saitama 350-1165, Japan; syunjirou-kobayashi@meg-snow.com (S.K.); jyamamura@meg-snow.com (J.Y.); s-higurashi@beanstalksnow.co.jp (S.H.)

**Keywords:** vitamin D, 25-hydroxyvitamin D, breast milk, LC-MS/MS, lifestyle

## Abstract

Background: Breast milk is considered the optimal source of nutrition during infancy. Although the vitamin D concentration in human breast milk is generally considered poor for infants, vitamin D in breast milk is an important source for exclusively breastfed infants. Increases in vitamin D insufficiency and deficiency in lactating mothers may reduce vitamin D concentrations in breast milk. This study aimed to compare vitamin D and 25-hydroxyvitamin D (25OHD) concentrations in breast milk collected in 1989 and 2016–2017 and simultaneously analyze them with liquid chromatography-tandem mass spectrometry (LC-MS/MS); the association between the lifestyle of recent lactating mothers (2016–2017) and vitamin D status in human breast milk was also evaluated. Method: Lactating mothers were recruited from three regions of Japan in 1989 (*n* = 72) and 2016–2017 (*n* = 90), and milk from 3–4 months was collected in summer and winter. The samples were strictly sealed and stored at −80℃ until measurement. Breast milk vitamin D and 25OHD concentrations were analyzed by LC-MS/MS. Vitamin D intake, sun exposure, and sunscreen use of the lactating mothers in 2016–2017 were assessed. Results: Both vitamin D and 25OHD concentrations in breast milk were higher in the summer regardless of the survey year. Significantly lower vitamin D and 25OHD concentrations were observed in 2016–2017 compared with 1989 in summer, but no survey year difference was observed in winter. The stepwise multiple regression analyses identified season, daily outdoor activity, and suntan in the last 12 months as independent factors associated with vitamin D_3_ concentrations. Conclusion: The results suggest that low vitamin D status in recent lactating mothers may have decreased vitamin D and 25OHD concentrations in breast milk compared with the 1980s. These results are helpful for developing public health strategies to improve vitamin D status in lactating mothers and infants.

## 1. Introduction

Vitamin D plays an important role in regulating calcium homeostasis and bone metabolism, and accumulating evidence has indicated that insufficient vitamin D levels are associated with many other health conditions, such as cancer, immune disorders, obesity, inflammation, hypertension, and mortality [1]. Recently, the high prevalence of vitamin D deficiency in both older and younger people has become a global problem [2]. Vitamin D deficiency in pregnant and lactating women is particularly problematic because it can affect not only their own bone metabolism but also fetal and infant growth via cord blood and breast milk. Parents are now advised to avoid direct sun exposure of their newborns [3], and there are increasing reports that exclusively breastfed infants with inadequate sunlight exposure and without vitamin D supplementation have an increased risk of rickets [4,5].

Although breast milk is considered the optimal source of nutrition during early infancy, the concentration of vitamin D in human milk is generally considered poor for infants [6]. The concentrations of vitamin D and 25-hydroxyvitamin D (25OHD) in human breast milk have been reported to range from 0.1–1.0 and 0.2–0.75 nmol/L, respectively [7,8,9,10,11]. However, there is no doubt that vitamin D in breast milk is an important source for infants who are exclusively breastfed, particularly in the early stages after birth. Since 1998 in Japan, the Maternal and Child Health Handbook has recommended outside air baths instead of sun baths. Although this policy has increased the importance of vitamin D in breast milk, it also lowered maternal awareness of the importance of vitamin D status for preventing rickets.

The serum 25OHD concentrations of breastfed infants without sun exposure are approximately 4 ng/mL lower at six weeks of age than at birth [12], and the vitamin D storage is exhausted at eight weeks [13]. Some studies have shown a positive correlation between the vitamin D status of lactating mothers and breast milk vitamin D and/or 25OHD concentrations [13,14,15,16]. Therefore, if lactating women develop a low vitamin D status, the vitamin D concentration in their breast milk will decrease, and their recipient infant will also be vitamin D-deficient. Unfortunately, a recent study reported that vitamin D deficiency in pregnant and lactating women was highly prevalent [17]. The number of children with symptomatic vitamin D deficiency has been increasing in recent years throughout the world [18,19,20,21], including in Japan [22,23,24,25,26]. Based on this discussion, the vitamin D concentration in breast milk associated with a decreased vitamin D status in the lactating mother may decrease over time and increase the risk of rickets. However, no previous studies have directly compared vitamin D or 25OHD concentrations from the breast milk of past and recent lactating mothers.

This study aimed to compare vitamin D and 25OHD concentrations in breast milk collected in 1989 and 2016–2017 that was analyzed simultaneously by liquid chromatography-tandem mass spectrometry (LC-MS/MS) and to evaluate the association between lifestyle factors of recent lactating mothers (in 2016–2017) and vitamin D or 25OHD concentrations in human breast milk. Understanding the changes of vitamin D concentrations in breast milk over time and its influencing factors can help create new suggestions for the prevention of vitamin D deficiency in infants.

## 2. Materials and Methods

### 2.1. Participants

This study was based on Japanese breast milk research projects conducted by Megmilk Snow Brand Co., Ltd. and Bean Stalk Snow Co., Ltd. at two different times: one study was performed in 1989 [27] and one study is a currently ongoing longitudinal prospective cohort study [28]. In both studies, lactating mothers were recruited from three regions (Kanto: 35–37° N, Kyusyu: 31–34° N, Hokkaido: 41–46° N). All subjects were non supplement user. Table 1 shows the age and number of lactating mothers in 1989 and 2016–2017. The participants in 1989 were significantly younger than those in 2016–2017 (age, 27.7 ± 3.8 vs. 30.3 ± 3.7 years). This tendency was observed in both summer and winter. No differences were observed in age when comparing the two seasons and three regions.

### 2.2. Breast Milk Samples

**Figure 1:**
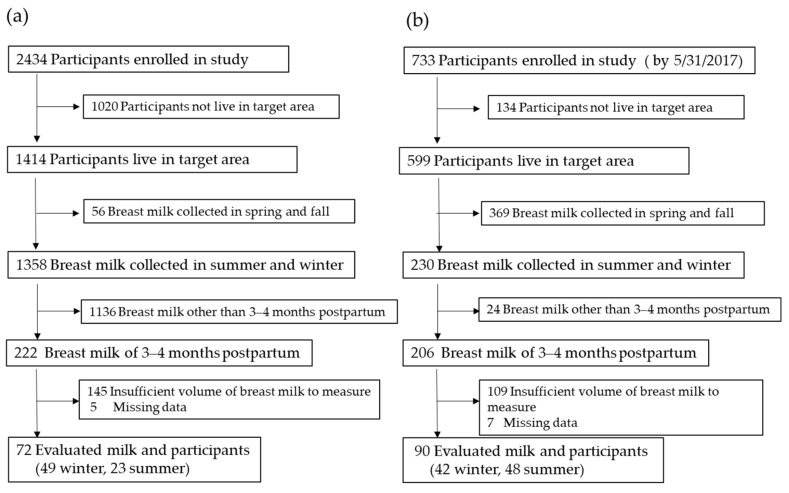
Participant and breast milk sample flow chart of the studies in 1989 (**a**) and 2016–2017 (**b**).

Breast milk samples (3–4 months postpartum), collected in the summer (July–September) and winter (January–March), were selected for measurement. Midmilk (1989) and mid-hindmilk (2016–2017) were manually collected by the mothers following directions in handed-out instructions. In the ongoing cohort study, we recruited participants who responded to an additional vitamin D-related questionnaire survey that was conducted from 2016 to 2017, as described below. As shown in the flow chart (Figure 1), 72 breast milk samples were collected in 1989 (winter, *n* = 49; summer, *n* = 23), and 90 samples were collected in 2016–2017 (winter, *n* = 42; summer, *n* = 48). The sample size was determined as sufficient through calculations using the G*Power 3.1.1 computer program software (Germany) [29]. A power analysis revealed that the number of participants was appropriate (with a power of 0.801 for both vitamin D and 25OHD). The amount and ages of the participants are summarized in Table 1. Approximately 50 mL of human breast milk was collected by manual expression at an intermediate time during suckling. The samples were strictly sealed and stored at −80 °C until being measured. Before extracting the vitamin D and 25OHD, the frozen breast milk was thawed and sonicated in ice water twice for 15 min.

### 2.3. Sample Preparation

The samples were prepared with some modifications of the method previously reported by Kamao et al. Each 10.0-mL breast milk sample was placed in a 50-mL screw-top vial. After the addition of 50 μL of internal standard solution (0.125 nmol/50 μL ethanol for *d*_7_-vitamin D_3_ and *d*_6_-25OHD_3_ [9,30]) and 20 mL of pyrogallolpared, with some modifications of the method previously reported by Kamao et al.(90%, *w*/*v*), the mixture was incubated at 90 °C for 180 min. Then, the mixture was transferred to a 200-mL separating funnel containing 40 mL of NaCl solution (1%, *w*/*v*), and fat-soluble matters were extracted twice with 30 mL of hexane-ethyl acetate (9:1, *v*/*v*), washed with water, and dehydrated with Na_2_SO_4_. The eluate was evaporated under reduced pressure, and the residue was dissolved with 1 mL of hexane. Furthermore, extracted unsaponifiable fat-soluble matter was applied to an InertSep SI FF column (500 mg/3 mL, GL Sciences, Inc. Tokyo, Japan), attached at the suction manifold (GL-SPE, GL Sciences, Inc. Tokyo, Japan), and washed with 4 mL of hexane and 2 mL of 2-propanol-hexane (5:95, *v*/*v*) to remove unnecessary fat-soluble matters. After washing, the fraction containing vitamin D_3_/D_2_ and 25OHD_3_/D_2_ was collected with 2 mL of 2-propanol-hexane (20:80, *v*/*v*).

4-[2-(6,7-dimethoxy-4-methyl-3-oxo-3,4-dihydroquinoxalyl)ethyl]-1,2,4-triazoline-3,5-dione (DMEQ-TAD) derivatization was performed according to the method of Higashi et al. [31]. The vitamin D and 25OHD fractions were dried and then dissolved with 150 μL of ethyl acetate containing DMEQ-TAD (0.4%, *w*/*v*). The mixture was kept at room temperature for 30 min, then an additional 150 μL of the same DMEQ-TAD reagent was added, and the entire mixture was kept at room temperature for another 1 h. After adding 1.5 mL of ethanol to decompose excess reagent, the mixture was kept at room temperature for 10 min and the solvent was evaporated. The residue was dissolved in 80 μL of acetonitrile, 30 μL of which was applied to LC-MS/MS. Standard solutions containing 6.25, 25, or 125 nmol/L of vitamin D_3_/D_2_ and 25OHD_3_/D_2_ with 125 nmol/L of the internal standard (*d*_7_-vitamin D_3_ and *d*_6_-25OHD_3_) were prepared and derivatized with DMEQ-TAD.

### 2.4. LC-MS/MS

Apparatus: The high performance liquid chromatography (HPLC) system consisted of a SCL-10ADvp system controller, two LC-10ADvp pumps, a DGC-14A automatic solvent degasser, a SIL-10ADvp auto injector, and a CTO-10ADvp column oven set to 35 °C (SHIMADZU Co., Kyoto, Japan). The HPLC system was coupled to an API 3000 triple-quadrupole tandem mass spectrometer (Applied Biosystems/MDS SCIEX, Foster City, CA, USA) equipped with an atmospheric pressure chemical ionization (APCI) source. Analyst (Ver. 1.4.2; Applied Biosystems/MDS SCIEX, Foster City, CA, USA) was used for data acquisition and analysis. Chromatographic and MS/MS conditions were described in a previous report [9]. All analytes were detected in the MS/MS-multiple reaction monitoring (MRM). The precursor and product ions (*m*/*z*) were as follows: DMEQ-TAD-vitamin D_3_ (*m*/*z*: 730.5/468.3), DMEQ-TAD-vitamin D_2_ (*m*/*z*: 742.6/468.3), DMEQ-TAD-25OHD_3_ (*m*/*z*: 746.5/468.1), DMEQ-TAD-25OHD_2_ (*m*/*z*: 758.5/468.2), DMEQ-TAD-*d*_7_-vitamin D_3_ (*m*/*z*: 737.6/468.2), and DMEQ-TAD-*d*_6_-25OHD_3_ (*m*/*z*: 752.5/468.1). The coefficients of variation (CVs) of the intra assay and inter assay of the pooled test breast milk were as follows: vitamin D_3_: 7.3% and 2.0% at 0.18 nmol/L; 25OHD_3_: 5.3% and 4.1% at 0.29 nmol/L; vitamin D_2_: 24.1% and 9.7% at 0.018 nmol/L; and 25OHD_2_: 43.8% and 5.5% at 0.012 nmol/L, respectively. The detection limit of all analytes was 0.001 nmol/L. For milk samples with concentrations below the detection limit, concentrations were used at half of the detection limit.

### 2.5. Lifestyle Parameters of the Lactating Women in 2016–2017

Vitamin D intake was estimated in the 2016–2017 lactating women using the brief-type self-administered diet history questionnaire (BDHQ) [32], which has been validated against 16-day dietary records. The BDHQ is a structured questionnaire that assesses dietary intake based on the reported consumption frequency of 58 different food and beverage items.

The participants in 2016–2017 also provided information on the status of sun exposure, sunscreen use, and vitamin D supplement use. These items were selected with reference to the Vitamin D & Sun (VIDSUN) questionnaire [33]. Additionally, the item “Paying attention to UV exposure” asked the subjects about their habits of using a parasol or wearing long-sleeve clothes for avoiding sun exposure. Moreover, information about the body weight and height of lactating mothers and newborns, gestational week, and parity were collected.

### 2.6. Calculation of Antirachitic Activity

One microgram of vitamin D is equivalent to 40 IU. Because orally consumed 25OHD has been shown to be five times more effective in raising circulating 25OHD concentrations than an equivalent amount of vitamin D [34], the antirachitic activity of 25OHD in breast milk was calculated as five times (1 µg = 200 IU) that of vitamin D [16].

### 2.7. Statistical Analyses

All statistical analyses were performed using the statistical software JMP Pro 14.0.0 (SAS Institute Inc., Cary, NC, USA). Student’s *t*-test was performed to determine the significance of difference in continuous variables between two groups (1989 vs. 2016–2017, summer vs. winter). One-way analysis of variance (one-way ANOVA) was performed to determine the significance of differences in continuous variables among more than two groups (regions, groups of frequency of sunscreen use). Simple regression analyses were performed to evaluate the association between continuous variable parameters and vitamin D or 25OHD concentrations in breast milk. Stepwise multiple linear regression analyses were performed to explore the determinants of vitamin D or 25OHD concentrations in breast milk. Significant variables detected in the simple linear regression analysis were included in the original model as plausible predictors. A forward stepwise regression was performed, and a *p* value > 0.10 was used for variable removal.

## 3. Results

### 3.1. Comparison of Breast Milk Vitamin D and 25OHD Concentrations between 1989 and 2016–2017

Table 2 shows the vitamin D_2_/D_3_ and 25OHD_2_/D_3_ concentrations in 1989 and 2016–2017. The vitamin D_2_ and 25OHD_2_ concentrations were significantly lower than the vitamin D_3_ or 25OHD_3_ concentrations in both 1989 and 2016–2017, and there were many non-detected (ND) samples in detection of vitamin D_2_ or 25OHD_2_. Therefore, we decided not to use vitamin D_2_ or 25OHD_2_ alone for analysis.

As shown in Table 2, vitamin D_3_ and 25OHD_3_ concentrations in 1989 tended to be higher than those in 2016–2017, but these differences were not statistically significant. Conversely, a significant difference in the total vitamin D concentration was observed between the two survey years (*p* = 0.044), whereas no significant difference was observed in the total 25OHD concentration. The calculated total antirachitic activities in breast milk from 1989 and 2016–2017 were 31.3 ± 30.3 and 25.5 ± 12.0 IU/L (mean ± SD), respectively.

**Figure 2:**
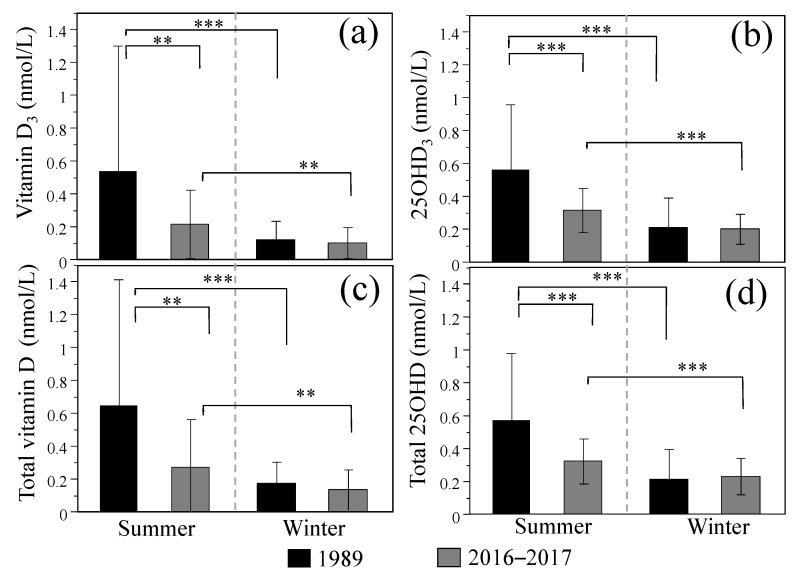
Breast milk vitamin D and 25-hydroxyvitamin D (25OHD) concentrations divided by season in 1989 and 2016–2017. Vitamin D_3_ (**a**), 25OHD_3_ (**b**), total vitamin D (**c**), and total 25OHD (**d**) concentrations in breast milk in the summer and winter. Solid bar: 1989, Gray bar: 2016–2017. Data are represented by the mean ± SD. Student’s *t*-test: ** *p* < 0.01, *** *p* < 0.001.

Figure 2 shows human breast milk vitamin D/D_3_ and 25OHD/D_3_ concentrations from 1989 and 2016–2017 divided by season. Both vitamin D_3_/total vitamin D and 25OHD_3_/total 25OHD concentrations in breast milk were higher in the summer, regardless of the survey year. In the summer, significantly lower vitamin D_3_/total vitamin D concentrations were observed in 2016–2017 (0.214 ± 0.208/0.269 ± 0.292 nmol/L) compared with 1989 (0.536 ± 0.764/0.646 ± 0.765 nmol/L), but no differences were observed between 2016–2017 (0.100 ± 0.095/0.133 ± 0.123 nmol/L) and 1989 (0.120 ± 0.111/0.175 ± 0.131 nmol/L) in the winter. Similarly, significantly lower 25OHD_3_/total 25OHD concentrations were observed in 2016–2017 (0.314 ± 0.135/0.323 ± 0.136 nmol/L) compared with 1989 (0.559 ± 0.399/0.569 ± 0.408 nmol/L) in the summer, but no difference was observed between 2016–2017 (0.200 ± 0.093/0.229 ± 0.111 nmol/L) and 1989 (0.211 ± 0.177/0.214 ± 0.181 nmol/L) in the winter. The calculated total antirachitic activities were as follows: summer: 55.5 ± 39.2 IU/L (1989) and 30.0 ± 12.5 IU/L (2016–2017) (*p* < 0.001); winter: 19.8 ± 15.4 IU/L (1989) and 20.4 ± 9.3 IU/L (2016–2017) (*p* = 0.828).

**Figure 3:**
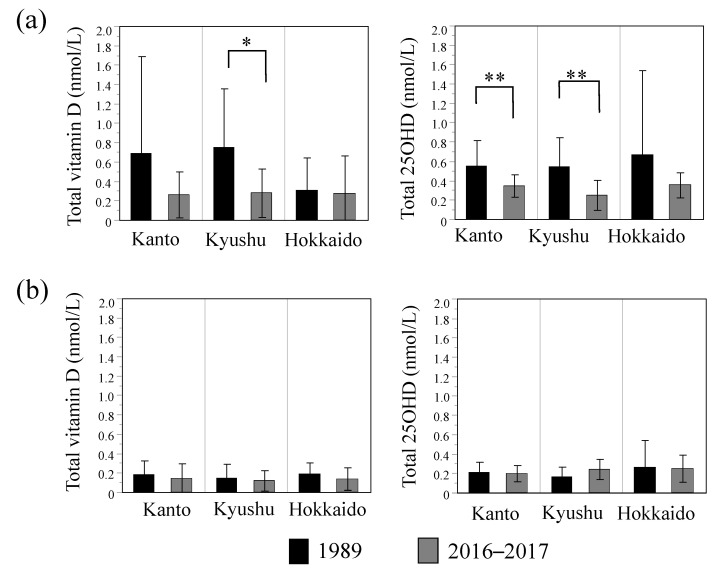
Breast milk vitamin D and 25OHD concentrations divided by season and region in 1989 and 2016–2017. Total vitamin D and Total 25OHD concentrations in summer (**a**) and winter (**b**). Solid bar: 1989, Gray bar: 2016–2017. Data are represented by the mean ± SD. Student’s *t*-test: * *p* < 0.05, ** *p* < 0.01.

Figure 3 shows the results of survey year comparison by region. In Kanto and Kyusyu, breast milk total 25OHD concentrations were significantly higher in 1989 than in 2016–2017, and a similar tendency was observed for total vitamin D concentrations in the summer. Conversely, in Hokkaido, which has the highest latitude among the three regions (41–46° N), no differences were observed in either vitamin D_3_ or 25OHD_3_ concentrations between 1989 and 2016–2017 for both seasons.

### 3.2. Effects of Lifestyle Parameters on Breast Milk Vitamin D and 25OHD Concentrations

Table 3 shows the background information of the lactating mothers in 2016–2017. Anthropometric parameters were in the normal range for Japanese women, and there were no abnormalities in their deliveries. The vitamin D intake of the lactating mothers was 11.9 ± 7.1 μg/day, which was higher than the adequate intake (AI) in the Dietary Reference of Intakes for Japanese, 2020 (8.5 μg/day), and 60.0% of lactating mothers had vitamin D intake more than the AI. The participants spent an average of 101.2 min/day on outdoor activities, and “more than 30 min” was the most frequent response for sunlight exposure time per day in the last one week. Approximately three-quarters of lactating mothers had experienced a suntan in the last 12 months. Conversely, half of the lactating mothers reported behaviors to avoid sunlight exposure and used sunscreen more than three days per week.

Table 4 shows the associations between lifestyle parameters and vitamin D_3_ or 25OHD_3_ concentrations in breast milk. Significant associations were observed between breast milk vitamin D_3_ concentrations and daily outdoor activity, season, or suntan in the last 12 months, and between breast milk 25OHD_3_ concentrations and season. Parameters that are not shown in Table 4 (maternal and newborn anthropometric parameters, gestational week, and parity) were not associated with either vitamin D_3_ or 25OHD_3_ concentrations. Age was unassociated with breast milk vitamin D_3_ and 25OHD_3_ concentrations. Although a similar analysis was performed for total vitamin D and total 25OHD concentrations, only the season was significantly associated with total vitamin D (*p* = 0.006) and total 25OHD (*p* < 0.001).

In the next analysis, stepwise multiple regression analyses were performed to explore the determinants of vitamin D_3_ and 25OHD_3_ concentrations in breast milk (Table 5). Daily outdoor activity, season, and suntan in the last 12 months were included as plausible predictors in the original model. The results indicated that daily outdoor activity, season, and suntan in the last 12 months were independently associated with breast milk D_3_ concentrations, and season was independently associated with breast milk 25OHD_3_ concentrations.

## 4. Discussion

To the best of our knowledge, this is the first study in which human breast milk vitamin D and 25OHD concentrations from relatively current lactating mothers and from 28 years ago were simultaneously measured with the LC-MS/MS method. This study identified a significant decrease of breast milk vitamin D concentrations in 2016–2017 compared with those in 1989 (Table 2). Obvious differences in both vitamin D and 25OHD concentrations between 2016–2017 and 1989 were observed in the summer but not in the winter (Figure 2). Although significant differences in age were observed between 2016–2017 and 1989, we confirmed that there was no correlation between age and breast milk vitamin D and 25OHD concentrations in both survey years.

No study has evaluated the stabilities of vitamin D and 25OHD in milk with a storage condition for 28 years at −80 °C in the dark. Vitamin D and 25OHD have been reported as stable compounds under light, heat, and oxygen exposures [35,36,37,38]. Renken et al. reported a slight loss of vitamin D_3_ in fortified milk upon exposure to light [35], but air exposure did not affect its stability in milk. Upreti et al. reported no detectable deterioration of vitamin D_3_ in fortified cheeses over 9 months at room and refrigerated temperatures [36]. However, Borai et al. reported the stability of 25OHD in serum at −80 °C for 6 months [37], and Colak et al. reported that exposure to room temperature for 4.5 h did not affect serum 25OHD measurements [38]. From the properties of both compounds, we can infer that the degradation of vitamin D and 25OHD in the samples in this study would be negligible or mild. Alternatively, in this study, mid-milk and mid-hindmilk were collected in 1989 and 2016–2017, respectively; við Streym et al. reported that both vitamin D and 25OHD levels were significantly higher in hindmilk than in foremilk [16]. Although foremilk was not used in both 1989 and 2016–2017, the samples obtained in 2016–2017 have a potential factor that shows higher vitamin D and 25OHD concentrations than those in samples obtained in 1989. However, contrary to this potential factor, breast milk vitamin D and 25OHD concentrations in the 2016–2017 samples were obviously lower than those in the 1989 samples.

Vitamin D deficiency is a worldwide problem, particularly in pregnant and lactating women. Yoshikata et al. reported a low serum 25OHD concentration in pregnant and lactating Japanese women living in the Kanto area [17]. They showed that serum 25OHD concentrations were 20.7–26.5 nmol/L during pregnancy and 32.8 nmol/L at the first month after delivery [17]. Shiraishi et al. reported that serum 25OHD concentrations of pregnant Japanese women in the summer (25.8 ± 12.8 nmol/L) were as low as those in the winter [39]. Unfortunately, we could not confirm a correlation with maternal vitamin D status because serum 25OHD concentrations could not be measured in our study; however, low vitamin D concentrations in breast milk in recent lactating mothers are predictive of their low vitamin D status.

We also confirmed that both vitamin D and 25OHD concentrations in breast milk were seasonally affected, and the phenomenon of higher concentrations in the summer than in the winter was similar, regardless of the survey year. These results were consistent with previous studies [14,16,40]; við Streym et al. reported that vitamin D or 25OHD concentration in breast milk of Danish lactating women were 0.9 or 1.6 nmol/L in summer, and 0.3 or 1.2 nmol/L in winter, respectively [16]. However, vitamin D or 25OHD concentration in our study were 0.27 or 0.32 nmol/L in summer and 0.13 or 0.23 nmol/L in winter, respectively, which were lower than those in the Danish study. Serum 25OHD concentration of Danish lactating mothers was reported as 73.6 nmol/L in summer and 55.5 nmol/L in winter. Although serum 25OHD concentration was not measured in our study, Yoshikata et al. reported that the recent annual average of serum 25OHD concentration in Japanese lactating women was 32.3 or 39.5 nmol/mL at 1 or 6 months postpartum, respectively [17]. These values in Japanese lactating mothers were lower than that in Danish lactating women, which strongly indicated the need for improvement of vitamin D status in Japanese lactating women.

One of the reasons why the concentration in breast milk in 2016–2017 was lower than that in 1989 may be the lifestyle of avoiding sunlight exposure in recent years. After the discovery of ozone layer depletion, it became well known that excessive exposure to solar radiation can increase the risk of skin cancer and photo-aging [41]; therefore, many people avoid exposure to solar radiation for health and cosmetic reasons. As a measure to address this, since 1998, the Ministry of Health, Labor, and Welfare in Japan, in the Maternal and Child Health Handbook, decided to recommend outside air baths, which do not need sunlight exposure, instead of sun baths. This policy should have highlighted the importance of vitamin D intake obtained from breast milk or formula milk. However, it lowered maternal awareness of the importance of vitamin D status for the prevention of rickets, and mothers began to focus on avoiding sunlight exposure to themselves and their babies. The fact that a significant difference in breast milk vitamin D concentrations was observed in the summer suggests that the behavior of avoiding sun exposure in lactating women affects the vitamin D concentration in breast milk. Moreover, in an analysis of the effects of lifestyle factors on breast milk vitamin D_3_ and 25OHD_3_ concentrations in 2016–2017, we confirmed that outdoor activity, season, and suntan in the last 12 months were independently associated with breast milk vitamin D_3_ concentrations, and season was independently associated with breast milk 25OHD_3_ concentrations (Table 5). This result also suggests that sunlight exposure has a significant effect on breast milk vitamin D_3_ and 25OHD_3_ concentrations.

We also confirmed significant secular differences in the summer total 25OHD concentrations in the Kanto and Kyushu areas, which had a relatively lower latitude, from Hokkaido (Figure 3). Similar tendencies were observed for total vitamin D concentrations, but a statistical difference was not obtained due to the high standard deviation in Kanto. One of the reasons why the data variation in breast milk vitamin D concentrations was larger than that of 25OHD may be the effect of differences in circulating stability. Wagner et al. reported that the half-life of vitamin D is 12–24 h, which is extremely short compared with that of 25OHD (2–3 weeks); furthermore, vitamin D preferentially enters breast milk compared with 25OHD [42]. Because the origin of vitamin D and 25OHD in breast milk is blood circulation, it is inferred that the difference in circulating kinetics was reflected in the variations of the concentration in breast milk. Because the sample number for summer in Hokkaido was small, further study with an increased sample number will be needed in this area.

This study did not identify a correlation between vitamin D intake assessed by the BDHQ and vitamin D and 25OHD concentrations in breast milk. However, this result does not indicate that maternal vitamin D intake does not affect breast milk vitamin D concentrations. It has previously been reported that vitamin D supplementation by the mother increases vitamin D and 25OHD concentrations in breast milk [43,44,45,46]. Takeuchi et al. reported that when 1200 IU (30 µg)/day of vitamin D_2_ were supplemented to lactating mothers for one month, breast milk vitamin D_2_ concentrations were elevated to 0.32 nmol/L [43]. Wagner et al. confirmed that when lactating women were supplemented with 6400 IU (160 µg)/day of vitamin D, the vitamin D concentration in breast milk increased to approximately 26 nmol/L after 1 month and 57 nmol/L after 6 months [44]. Moreover, Dawodu et al. reported that supplementation of 6000 IU (150 µg)/day in lactating women increased vitamin D concentrations in breast milk to 185 IU/L [46]. The results of these studies with high-dose vitamin D supplementation clearly show a positive correlation between vitamin D intake and breast milk vitamin D concentrations. Conversely, Hollis et al. reported that, with limited sun exposure, an intake of 400 IU (10 µg)/day of vitamin D would not sustain maternal circulating 25OHD concentrations and would supply only limited amounts of vitamin D to nursing infants via breast milk [47]. The vitamin D intake of lactating women in our study was 11.9 ± 7.1 µg/day, which is approximately equivalent to the intake reported by Hollis et al. that does not affect breast milk concentrations.

This study is limited by the fact that we could not measure the serum 25OHD concentrations of the lactating mothers, and by the lack of lifestyle data from 1989. Another limitation of this study is that the results were obtained from a limited number of Japanese lactating women. However, the strength of this study is that relatively recent and past breast milk samples collected under similar conditions were used in the simultaneous measurements.

## 5. Conclusions

Our results suggest that the recent low vitamin D status in lactating mothers may result in decreased vitamin D and 25OHD concentrations in breast milk compared with the 1980s. The decrease of vitamin D concentrations in breast milk appeared in the summer, which suggests that the lifestyle of avoiding sunlight exposure may affect the vitamin D concentration in breast milk. We also confirmed that not only season but also daily outdoor activity and suntan in the last 12 months independently affected breast milk vitamin D_3_ concentrations in 2016–2017. Breast milk is essentially the sole source of vitamin D for infants with minimal sunlight exposure. The sample size of our study was relatively small, but the results were significant. Our findings strongly suggest the need to improve maternal vitamin D status to prevent rickets. These results can be useful for developing public health strategies to improve vitamin D status in lactating mothers and infants not only in Japan but also in other countries where the importance of vitamin D status is not emphasized to lactating women.

## Figures and Tables

**Table 1:** Number and age of lactating mothers in 1989 and 2016–2017.

Participants	Total			Regions								
				Kanto (35–37° N)			Kyushu (31–34° N)			Hokkaido (41–46° N)		
	N	Age		N	Age		N	Age		N	Age	
<1989>												
All	72	27.7	±3.8	28	26.8	±3.7	24	28.0	±3.5	20	28.8	±4.2
Summer	23	27.3	±4.3	10	26.2	±4.8	9	28.1	±3.7	4	28.5	±4.4
Winter	49	27.9	±3.6	18	27.2	±3.0	15	27.9	±3.6	16	28.8	±4.3
<2016~2017>												
All	90	30.3	±3.7	34	30.5	±3.6	28	30.3	±3.5	28	30.3	±4.2
Summer	48	30.2	±3.6	20	31.1	±3.6	13	29.6	±3.0	15	29.6	±4.1
Winter	42	30.5	±3.9	14	29.6	±3.7	15	30.8	±4.0	13	31.1	±4.3

Data are represented by Mean ± SD.

**Table 2:** Vitamin D and 25OHD concentrations in human breast milk.

Survey Year	Vitamin D_3_	Vitamin D_2_	Total Vitamin D	25OHD_3_	25OHD_2_	Total 25OHD
	Mean ± SD	Mean ± SD	Mean ± SD	Mean ± SD	Mean ± SD	Mean ± SD
<1989>	0.252 ± 0.477	0.073 ± 0.120	0.325 ± 0.492	0.322 ± 0.312	0.006 ± 0.014	0.328 ± 0.319
	ND: 5%	ND: 22%		ND: 0%	ND: 82%	
<2016–2017>	0.161 ± 0.174	0.045 ± 0.138	0.206 ± 0.238	0.261 ± 0.130	0.018 ± 0.035	0.279 ± 0.133
	ND: 0%	ND: 3.2%		ND: 4.4%	ND: 16%	
*p* *	0.094	―	0.044	0.094	―	0.191

* Student’s *t* test, (nmol/L); ND: not detected.

**Table 3:** Background characteristics of lactating mothers in 2016–2017.

Parameters	Mean (SD) or N (%)	
Maternal height (cm)	158.4	(5.3)
Maternal body weight (kg)	54.1	(6.9)
Maternal body mass index (BMI: kg/m^2^)	21.6	(2.7)
Parity (times)	1.5	(0.5)
Vitamin D intake (μg/day)	11.9	(7.1)
Daily outdoor activity (min/day)	101.2	(70.8)
Suntan in the last 12 months [Yes]	21	(23%)
Behavior to avoid sunlight exposure [Yes] (e.g., using parasol or walking in the shade)	45	(50%)
Frequency of sunscreen use per week		
1: 7 days	18	(20%)
2: 5–6 days	16	(18%)
3: 3–4 days	9	(10%)
4: 1–2 days	20	(22%)
5: 0 day	27	(30%)
Sunlight exposure time per day in the last one week		
1: >30 min	39	(43%)
2: 15–30 min	27	(30%)
3: 5–15 min	17	(19%)
4: <5 min	7	(8%)

BMI, body mass index; e.g., exempli gratia.

**Table 4:** Association between parameters and vitamin D_3_ or 25OHD_3_ concentration in breast milk.

Parameters	Vitamin D_3_ Concentration			25OHD_3_ Concentration		
	*β*	*R^2^*	*P*	*β*	*R^2^*	*P*
Age (years) ^a^	−2.6 × 10^−3^	0.003	0.600	1.7 × 10^−3^	0.002	0.649
BMI (kg/m^2^) ^a^	3.8 × 10^−4^	<0.001	0.956	2.7 × 10^−3^	0.003	0.605
Vitamin D intake (µg/day) ^a^	2.1 × 10^−3^	0.007	0.429	−5.3 × 10^−4^	0.001	0.784
Daily outdoor activity (min) ^a^	6.0 × 10^−4^	0.059	0.021	1.4 × 10^−4^	0.006	0.470
Season [Winter-Summer] ^b^	−0.113	0.107	0.002	−0.115	0.196	<0.001
Suntan in last 12 months [Yes-No] ^b^	0.107	0.069	0.012	−0.007	0.001	0.824
Regions [Kanto, Kyusyu, Hokkaido] ^b^	—	0.008	0.697	—	0.045	0.135
Frequency of sunscreen use per week [1: 7 days, 2: 5–6 days, 3: 3–4 days, 4: 1–2 days, 5: 0 days] ^b^	—	0.043	0.433	—	0.055	0.298

^a^ Simple regression analysis, ^b^ Analysis of variance, BMI, body mass index.

**Table 5:** Stepwise multiple regression analysis for parameters associated with vitamin D_3_ or 25OHD_3_ concentration in breast milk.

Parameters	Vitamin D_3_ Concentration			25OHD_3_ Concentration		
	*β*	*R^2^*	*P*	*β*	*R^2^*	*P*
Season [Winter-Summer]	−0.050	0.107	0.002	−0.060	0.196	<0.001
Daily outdoor activity time (min)	6.0 × 10^−4^	0.059	0.015	1.5 × 10^4^	0.006	0.410
Suntan in last 12 months [Yes-No]	−0.042	0.040	0.040	−0.018	0.013	0.232
